# Smoking Cessation Reverses DNA Double-Strand Breaks in Human Mononuclear Cells

**DOI:** 10.1371/journal.pone.0103993

**Published:** 2014-08-05

**Authors:** Mari Ishida, Takafumi Ishida, Satoshi Tashiro, Hitomi Uchida, Chiemi Sakai, Naoya Hironobe, Katsuya Miura, Yu Hashimoto, Koji Arihiro, Kazuaki Chayama, Yasuki Kihara, Masao Yoshizumi

**Affiliations:** 1 Department of Cardiovascular Physiology and Medicine, Hiroshima University Graduate School of Biomedical and Health Sciences, Hiroshima, Japan; 2 Department of Cardiovascular Medicine, Hiroshima University Graduate School of Biomedical and Health Sciences, Hiroshima, Japan; 3 Department of Cellular Biology, Research Institute for Radiation Biology and Medicine, Hiroshima University, Hiroshima, Japan; 4 Department of Anatomical Pathology, Hiroshima University Hospital, Hiroshima, Japan; 5 Department of Gastroenterology and Metabolism, Hiroshima University Graduate School of Biomedical and Health Sciences, Hiroshima, Japan; Tulane University Health Sciences Center, United States of America

## Abstract

**Objective:**

Cigarette smoking is a major risk factor for atherosclerotic cardiovascular disease, which is responsible for a significant proportion of smoking-related deaths. However, the precise mechanism whereby smoking induces this pathology has not been fully delineated. Based on observation of DNA double-strand breaks (DSBs), the most harmful type of DNA damage, in atherosclerotic lesions, we hypothesized that there is a direct association between smoking and DSBs. The goal of this study was to investigate whether smoking induces DSBs and smoking cessation reverses DSBs *in* *vivo* through examination of peripheral mononuclear cells (MNCs).

**Approach and Results:**

Immunoreactivity of oxidative modification of DNA and DSBs were increased in human atherosclerotic lesions but not in the adjacent normal area. DSBs in human MNCs isolated from the blood of volunteers can be detected as cytologically visible “foci” using an antibody against the phosphorylated form of the histone H2AX (γ-H2AX). Young healthy active smokers (n = 15) showed increased γ-H2AX foci number when compared with non-smokers (n = 12) (foci number/cell: median, 0.37/cell; interquartile range [IQR], 0.31–0.58 vs. 4.36/cell; IQR, 3.09–7.39, p<0.0001). Smoking cessation for 1 month reduced the γ-H2AX foci number (median, 4.44/cell; IQR, 4.36–5.24 to 0.28/cell; IQR, 0.12–0.53, p<0.05). A positive correlation was noted between γ-H2AX foci number and exhaled carbon monoxide levels (r = 0.75, p<0.01).

**Conclusions:**

Smoking induces DSBs in human MNCs *in* *vivo*, and importantly, smoking cessation for 1 month resulted in a decrease in DSBs to a level comparable to that seen in non-smokers. These data reinforce the notion that the cigarette smoking induces DSBs and highlight the importance of smoking cessation.

## Introduction

Cigarette smoking is a major risk factor for atherosclerotic cardiovascular disease. When compared with non-smokers, smokers are twice as likely to experience acute myocardial infarction [1], which contributes to the increase in mortality seen in smokers [2]. The molecular mechanisms by which smoking promotes atherosclerosis have not been fully delineated. Cigarette smoke contains thousands of chemicals and compounds, including many oxidants and free radicals that induce oxidative damage. Further, cigarette smoke causes oxidative damage in DNA, either directly or through generation of reactive oxygen species in cultured cells [3].

While most DNA damage caused by reactive oxygen species, chemicals, radiation, and other noxious stimuli is efficiently repaired by specific DNA repair systems, persistence and accumulation of DNA damage can occur depending on the severity of the damage and the capacity of DNA repair mechanisms. Among several types of DNA damage, double-strand breaks (DSBs) are the most serious form of DNA damage and are difficult to repair accurately [4]. Accumulated DNA damage results in mutagenesis and chromosomal rearrangements, leading to genomic instability.

Several recent studies suggest that DNA damage is involved in the pathogenesis of atherosclerosis. First, patients with progeroid syndromes (e.g., Werner syndrome), which arise from abnormalities in DNA repair and subsequent DNA damage, develop atherosclerotic disease at a young age [5]. Second, exposure of the heart to ionizing radiation during radiotherapy for breast cancer increases the risk of coronary artery disease [6].

There are several studies of the link between smoking and DSBs formation [7]–[10]. Most of these studies are *in* *vitro* experiments showing that cigarette smoke induces DSBs formation in cultured cells. However, it remains uncertain whether smoking induces DSBs *in* *vivo*. When DSBs accumulate without repairing accurately, cellular senescence or apoptosis occurs, which is observed in atherosclerotic lesions [11].

To further characterize the pathogenic link between cigarette smoke and cardiovascular disease, this study investigated whether DSBs are present in human atherosclerotic plaque and assessed the effect of cigarette smoking and the effect of smoking cessation on the level of DSBs in human cells *in* *vivo*.

## Materials and Methods

### Immunohistochemistry

Immunohistochemistry was performed on tissue sections obtained from autopsy. Written informed consent from the next of kin was obtained for use of the tissue samples in research, in accordance with the ethical committee of Hiroshima University. Anti-phospho-histone H2AX (γ-H2AX) antibody was applied overnight at 4°C, biotinylated secondary antibody was incubated for 1 hour at 20°C, and diaminobenzidine (DAB) was used as the chromogen. Staining specificity was assessed by omitting the primary antibody.

### Cell culture

Human aortic smooth muscle cells and human umbilical vein endothelial cells were purchased from CAMBREX corporation and cultured in basal medium containing the specific growth supplements recommended by the manufacturer. Cells at passage 4 to 8 were used.

### Western blot analysis

Western blot was performed as described previously [12].

### Human subjects

Twenty-seven young healthy volunteers (12 male non-smokers, 15 male active smokers; age: 24.3±0.5 years) were included in the first portion of the study. The second portion of this study included six male smokers who intended to quit smoking (age: 38.3±5.1 years). The study protocol was approved by the Hiroshima University Institutional Review Board (approval number, M746-2). Written informed consent was obtained from all subjects.

Blood was drawn from a peripheral vein after an overnight fast. Laboratory-based measurements included plasma cholesterol, triglycerides, high-density lipoprotein cholesterol concentrations, low-density lipoprotein cholesterol concentrations, plasma glucose, hemoglobin A1c, and uric acid. For protocol 2, blood was drawn before and 1 month after smoking cessation. Exhaled carbon monoxide (CO) was measured using a portable CO analyzer (Smokerlyzer, Bedfont Scientific, England) and was used as an objective measure of the number of cigarettes smoked per day [13], [14] and to assess smoking cessation status. CO readings >6 ppm strongly suggests that the subject is actively smoking [14].

### Immunofluorescent analysis

Circulating mononuclear cells (MNCs), which are easily obtained from peripheral blood and are therefore a good cell-type for investigation of in vivo endpoints, were isolated using Lymphoprep. Mononuclear cells were isolated by centrifugation according to manufacturer’s instruction and were placed on glass slides for subsequent immunofluorescent analysis. Cells were fixed with 4% paraformaldehyde, and nuclei were permeabilized with Triton X-100. Cells were then incubated with anti-γ-H2AX antibody for 30 min at 37°C, followed by incubation with Cy3-conjugated secondary antibodies for 30 min at 37°C. Nuclei were stained with Hoechst 33342.

Cells were assessed with an Axioplan2 microscope with AxioCam MR controlled by Axiovision software (Zeiss, Thornwood, NY), and γ-H2AX foci were counted in at least 100 cells for each specimen. These cytologically visible foci were used as an indicator of DSBs. γ-H2AX foci numbers were divided by the total cell number and are expressed as the γ-H2AX foci number/cell. γ-H2AX foci-positive cells were also counted, divided by the total cell number, and are expressed as a percentage of γ-H2AX foci-positive cells.

### Statistical analysis

Data are expressed as medians and interquartile range (IQR). The Mann-Whitney U test was used to determine statistical differences between the two groups. Paired comparisons of DNA damage before and after smoking cessation were performed using the Wilcoxon signed-rank test. A *P* value less than 0.05 was considered to indicate statistical significance. The relationship between breath CO and γ-H2AX foci number in MNCs was assessed by Spearman's rank-correlation test.

## Results

### DSBs are present in human atherosclerotic plaques

**Figure 1:**
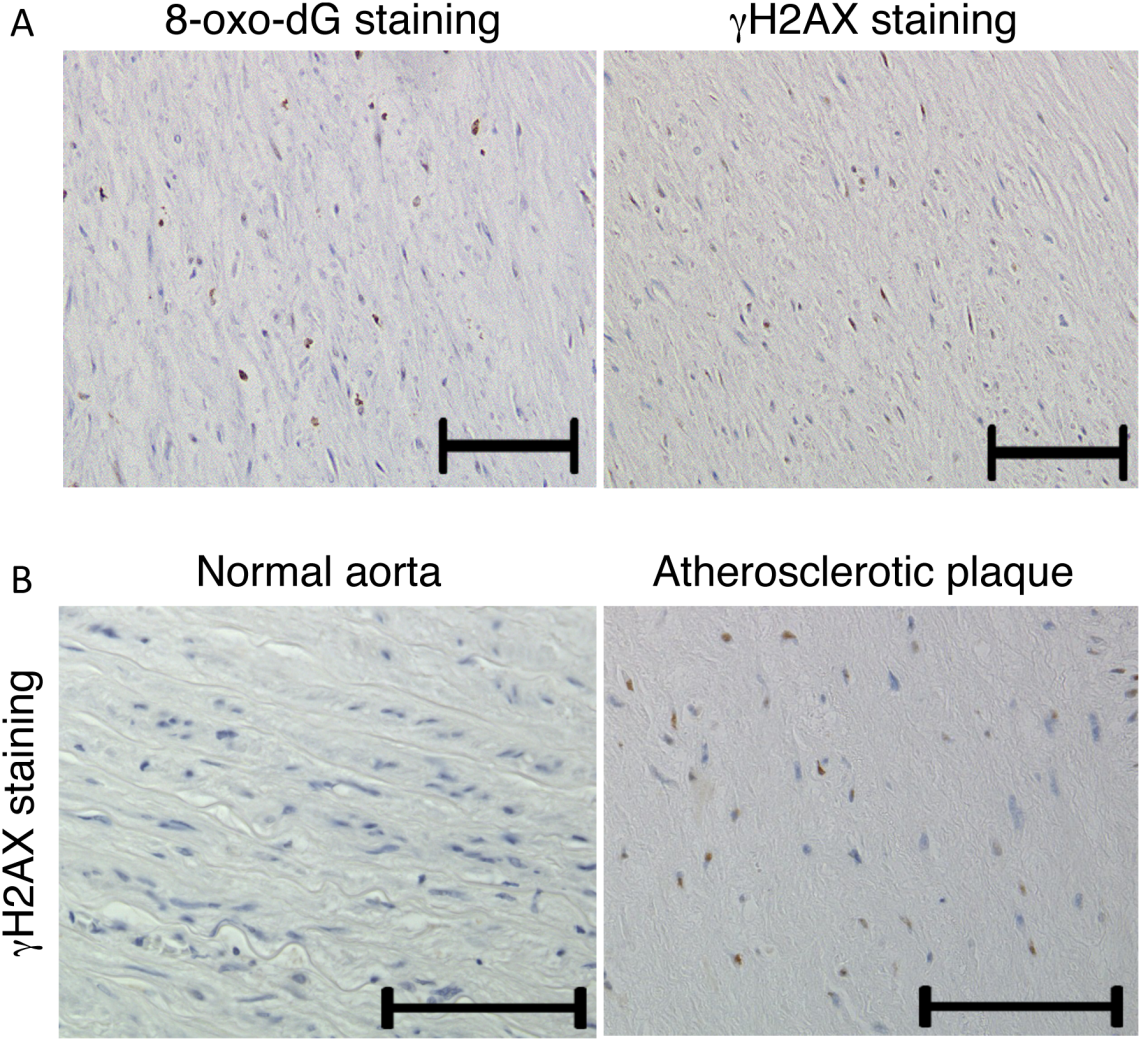
DNA damage is present in human atherosclerotic plaque. A, Co-localization of 8-oxo-dG and γ-H2AX in atherosclerotic lesion. B, The presence of γ-H2AX foci in atherosclerotic lesions from autopsy detected by immunohistochemical staining for γ-H2AX. No staining was detected in the normal vessel. Bar = 100 µm.

To investigate whether DNA damage and repair is involved in the pathogenesis of atherosclerosis, atherosclerotic lesions obtained from autopsy were evaluated by immunohistochemistry. It is well known that reactive oxygen species (ROS) induce oxidative modification of DNA [i.e., the formation of 7,8-dihydro-8-oxo-2′-deoxyguanosine (8-oxo-dG)]. As expected, increased immunoreactivity against 8-oxo-dG was observed in atherosclerotic lesions (Fig. 1A).

When DSBs occur, histone H2AX around the DSBs is phosphorylated, and phosphorylated H2AX (γ-H2AX) triggers downstream DNA damage response (DDR) [15]. Thus, γ-H2AX is a key player in DDR. Immunoreactivity against γ-H2AX, which is a sensitive marker for DSBs, was also increased in human atherosclerotic lesions but not in the adjacent normal area (Fig. 1B). γ-H2AX staining was observed in almost the same region at which 8-oxo-dG immunoreactivity was observed in sequential sections (Fig. 1A).

### H_2_O_2_ induces phosphorylation of histone H2AX

**Figure 2:**
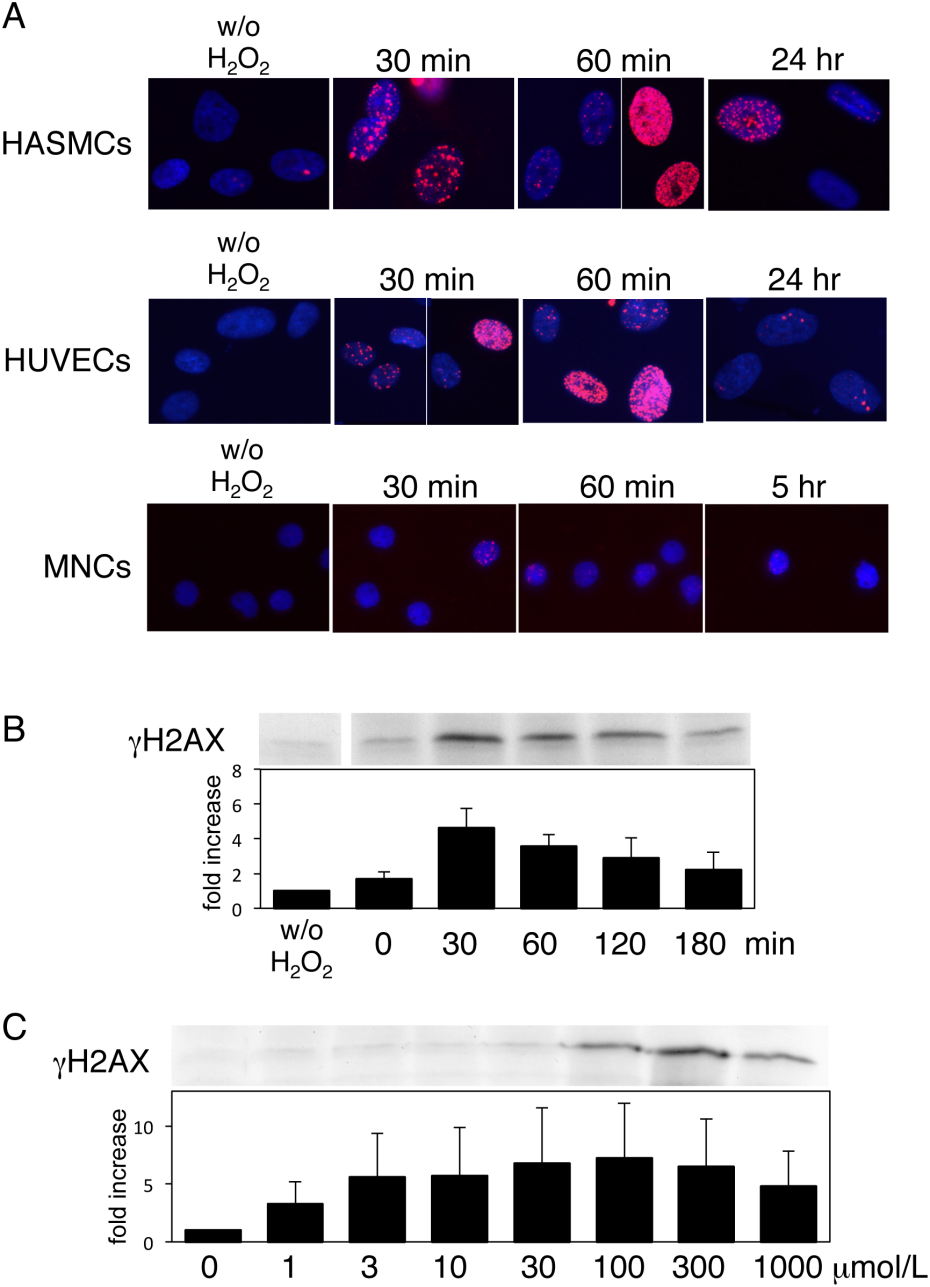
Oxidative stress induces foci formation of γ-H2AX in human vascular cells and human peripheral mononuclear cells (MNCs). A, Immunofluorescent staining of the γ-H2AX (red) in human aortic smooth muscle cells (HASMCs), human umbilical vein endothelial cells (HUVECs), and MNCs. Cells were treated with H_2_O_2_ for 15 minutes, moved to normal medium, and fixed at each time point after medium change as indicated. γ-H2AX immunostaining was performed as described in “Materials and methods”. Representative data are shown. B, Time course of γ-H2AX formation by Western blot analysis. Cells were treated with H_2_O_2_ for 15 minutes, moved to normal medium, and harvested at each time point as indicated. Western blotting using γ-H2AX antibody was performed as described in Materials and methods. Representative data are shown. Averages of five independent experiments are shown in the graph (Data are expressed mean ± standard error). C, H_2_O_2_ dose dependency of γ-H2AX formation by Western blot analysis. Cells were treated with various concentrations of H_2_O_2_ for 15 minutes, moved to normal medium, and harvested at 30 min after medium change. Western blotting using γ-H2AX antibody was performed as described in “Materials and methods”. Representative data are shown. Averages of three independent experiments are shown in the graph (Data are expressed mean ± standard error).

Jorgensen et al. reported that smoke from nicotine-free cigarettes induced DDR in A549 pulmonary adenocarcinoma cells and that the DDR was greatly reduced by the free radical scavenger, N-acetyl-L-cysteine [9]. Thus, we investigated whether oxidative stress directly induces the formation of DSBs in vascular cells. H_2_O_2_-induced γ-H2AX foci formation was detected by immunofluorescent studies with γ*-*H2AX antibody (Fig. 2A) in human aortic smooth muscle cells and human umbilical vein endothelial cells. γ-H2AX foci were observed in almost 100% of the cell nuclei after H_2_O_2_ treatment. Western blotting with γ-H2AX antibody showed that DSBs were induced by H_2_O_2_ with peak at 30 minutes after being moved to normal medium (Fig. 2B). The γ-H2AX foci formation was H_2_O_2_ dose-dependent and was induced by as little as 1 µmol/L H_2_O_2_ (Fig. 2C). Immunofluorescent studies showed that the extent of damage and the repair varied among the individual cells; while some cells were quickly repaired at 60 minutes, some cells could not be repaired even after 24 hours, and these cells showed persistent DNA damage. Similarly, H_2_O_2_ also induces DSBs in MNCs (Fig. 2A). These results suggest that oxidative stress directly induces DNA DSBs in cultured vascular cells and MNCs.

### DSBs in MNCs of active smokers and non-smokers

**Figure 3:**
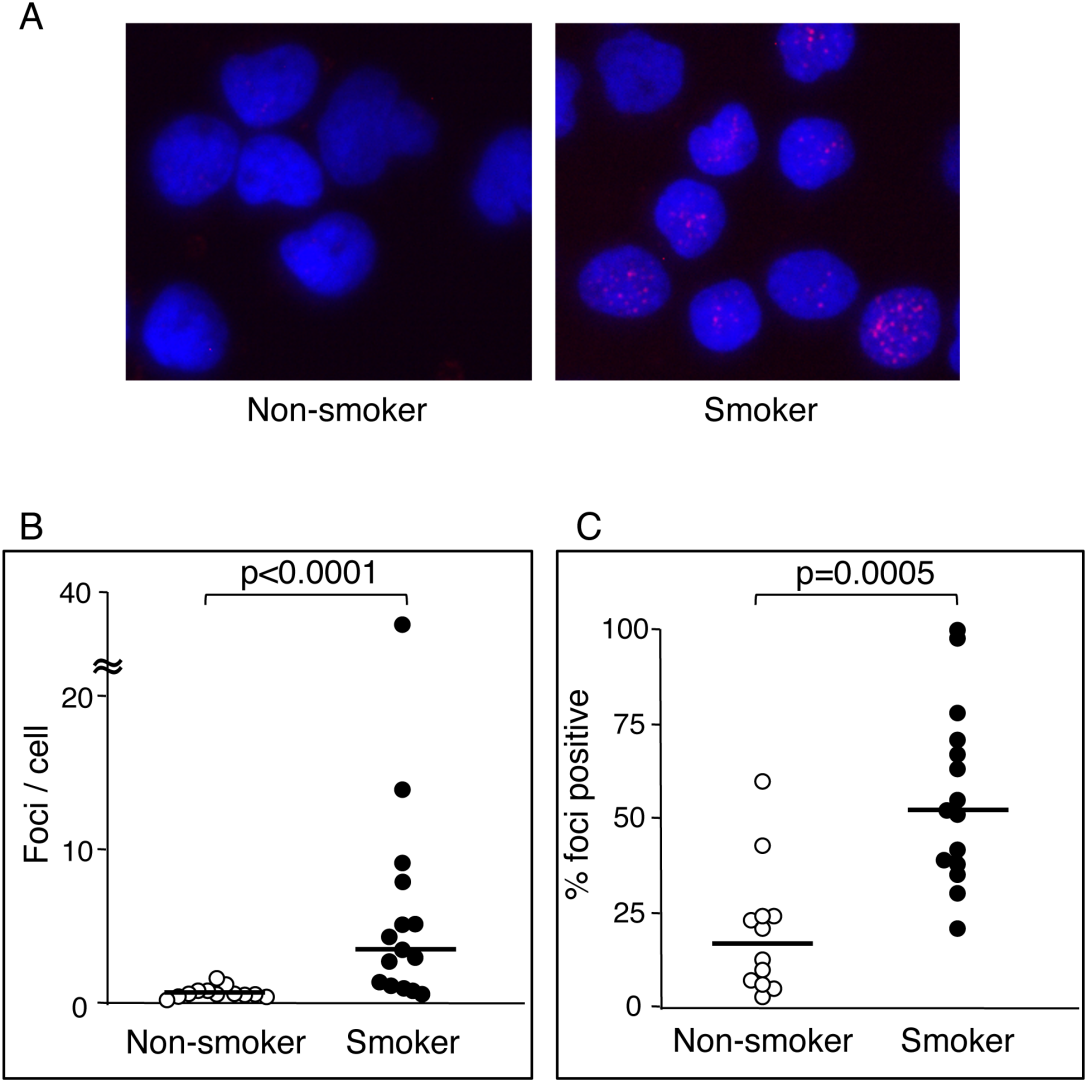
Double-strand breaks in mononuclear cells of non-smokers and smokers. A, Representative images of γ-H2AX foci (red) in mononuclear cells from a non-smoker and a smoker. B and C, Differences in foci number per cell (B) and percentage of foci positive cells (C) between young healthy smokers and non-smokers. The Mann-Whitney U test was used to determine statistical differences between the two groups. A P value less than 0.05 was considered as statistically significant. The line across the dot plot identifies the median sample value.

We next examined whether cigarette smoking induces DSBs in human MNCs *in* *vivo* as a surrogate marker for DNA damage of vascular cells. Twenty-seven young healthy volunteers (12 male non-smokers, 15 male active smokers; age: 24.3±0.5 years) were included. Baseline characteristics of young healthy smokers and non-smokers are shown in Table 1. None of the measured variables were significantly different between the two groups, with the exception of pack-years. The γ-H2AX foci number/cell was significantly higher in smokers (median 4.36/cell; IQR, 3.09–7.39) than in non-smokers (median 0.37/cell; IQR, 0.31–0.58) (p<0.0001) (Fig. 3A). The percentage of γ-H2AX foci-positive cells was also significantly higher in smokers than in non-smokers (54.9%; IQR, 37.8–67.0 vs. 17.0%; IQR, 10.7–24.0, respectively, p = 0.0005) (Fig. 3B). This result suggests that smoking causes DSBs *in* *vivo*, as assessed by characterization of γ-H2AX foci.

**Table 1:** Baseline characteristics of the study participants.

	Non-smokers (n = 12)		Smokers (n = 15)		p
	Median	IQR	Median	IQR	
Age, years	23.5	23–24	24	23–25.8	NS
Pack year (pack/day x years)	0		4	3.5–6.8	<0.001
BMI, kg/m^2^	22.3	21.7–22.9	20.3	19.4–23.7	NS
SBP, mmHg	110	110–123	118	114–122	NS
DBP, mmHg	70	68–74	76	70–80	NS
Fasting blood glucose, mg/dL	87	86–90	78	78–81	NS
Uric acid, mg/dL	6.2	6.0–6.4	6.2	5.8–7.3	NS
Triglycerides, mg/dL	57	51–65	105	85–106	NS
HDL cholesterol, mg/dL	64	58–73	44	42–61	NS
LDL cholesterol, mg/dL	84	84–84.3	78	75–112	NS

Data are median and interquartile range (IQR). A *P* value less than 0.05 was considered to indicate statistical significance based on the Mann-Whitney U test.

Abbreviations: BMI, body mass index (calculated as weight in kilograms divided by height in meters squared); SBP, systolic blood pressure; DBP, diastolic blood pressure; HDL, high-density lipoprotein; LDL, low-density lipoprotein; NS, not significant.

### Effect of smoking cessation on DSBs

We next examined the effect of smoking cessation on DSBs in MNCs. The γ-H2AX foci number in MNCs was examined in participants before and after smoking cessation. Six male smokers who intended to quit smoking were included. Characteristics of the subjects before and after smoking cessation are shown in Table 2. These subjects included one patient with diabetes and one patient with high serum triglycerides; the other subjects were healthy. Medical regimens used to treat comorbid conditions in these subjects remained unchanged throughout this study, including during the smoking cessation period. The median exhaled CO value was 16 ppm (IQR, 9–20 ppm) before smoking cessation and 2 ppm (IQR, 2–2.5) after 1 month of smoking cessation (less than 3 ppm in all subjects after smoking cessation), which suggests that smoking cessation efforts were successful.

**Table 2:** Baseline characteristics of the participants.

	Before smoking cessation		After smoking cessation		p
	Median	IQR	Median	IQR	
Age, years	37.5	28.3–44.5			-
Pack year (pack/day x years)	17	8.5–25.2	-		-
Breath CO, ppm	16	9–20	2	2–2.5	0.02
BMI, kg/m^2^	22	20.5–24	21.9	20–24.3	NS
SBP, mmHg	120	112–127	120	110–128	NS
DBP, mmHg	63	60–75	65	59–73	NS
Fasting blood glucose, mg/dL	95.5	91.3–98.3	98.0	98.0–102	NS
Uric acid, mg/dL	7.1	6.2–7.7	6.3	6.2–7.1	NS
Triglycerides, mg/dL	141	91–447	161	147–211	NS
HDL cholesterol, mg/dL	53	46–60	58	50–58	NS
LDL cholesterol, mg/dL	102	88–114	119	115–125	NS

Data are median and interquartile range (IQR). A *P* value less than 0.05 was considered to indicate statistical significance based on the Wilcoxon signed-rank test.

Abbreviations: CO, exhaled carbon monoxide; BMI, body mass index (calculated as weight in kilograms divided by height in meters squared); SBP, systolic blood pressure; DBP, diastolic blood pressure; HDL, high-density lipoprotein; LDL, low-density lipoprotein; NS, not significant.

**Figure 4:**
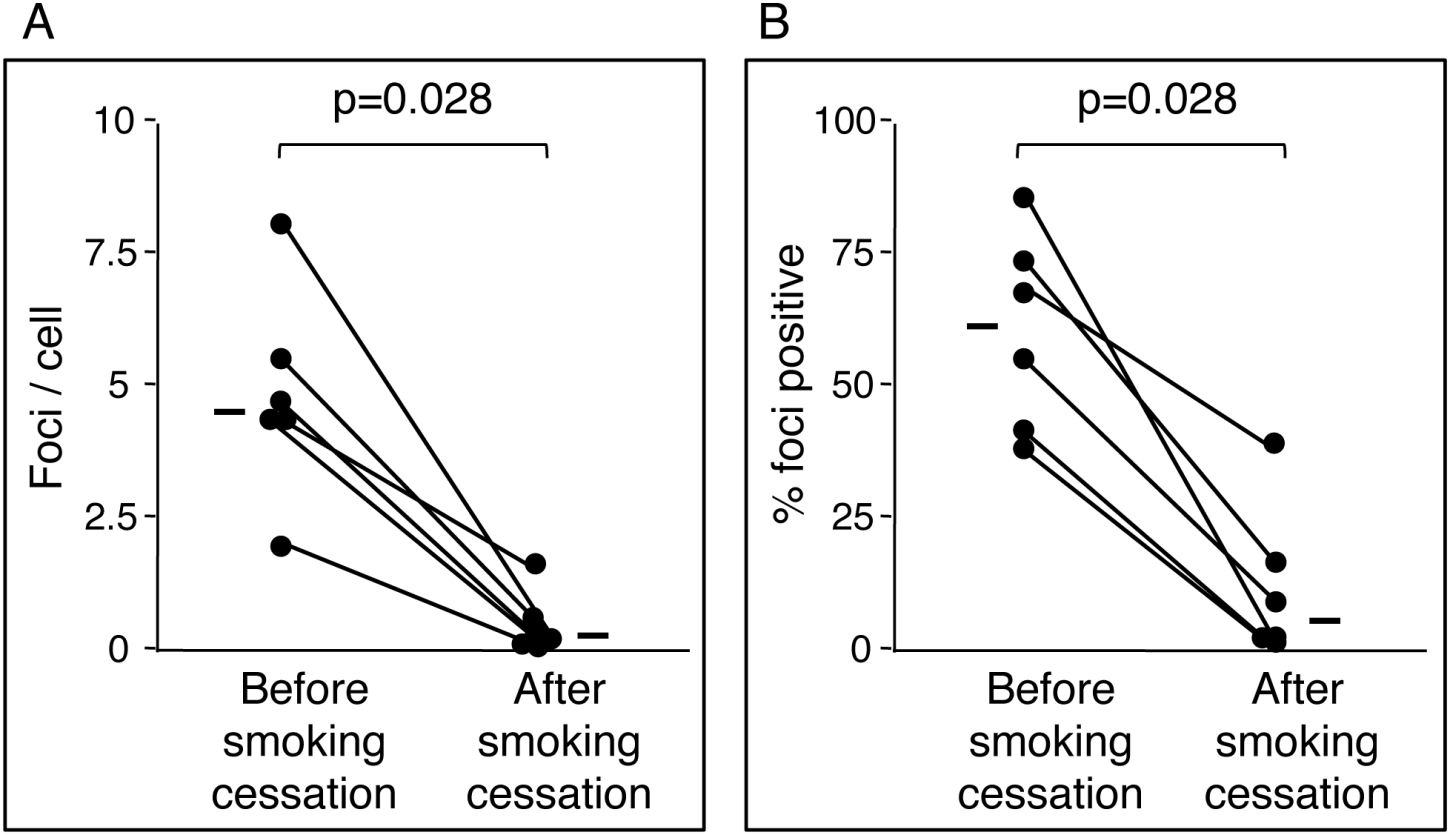
Effect of smoking cessation on double-strand breaks in mononuclear cell. Changes in foci number per cell (A) and changes in percentage of foci positive cells (B) in response to smoking cessation. The Wilcoxon signed-rank test was used to determine statistical differences for paired comparisons of DNA damage before and after smoking cessation. A P value less than 0.05 was considered as statistically significant. The line beside the dot plot identifies the median sample value.

**Figure 5:**
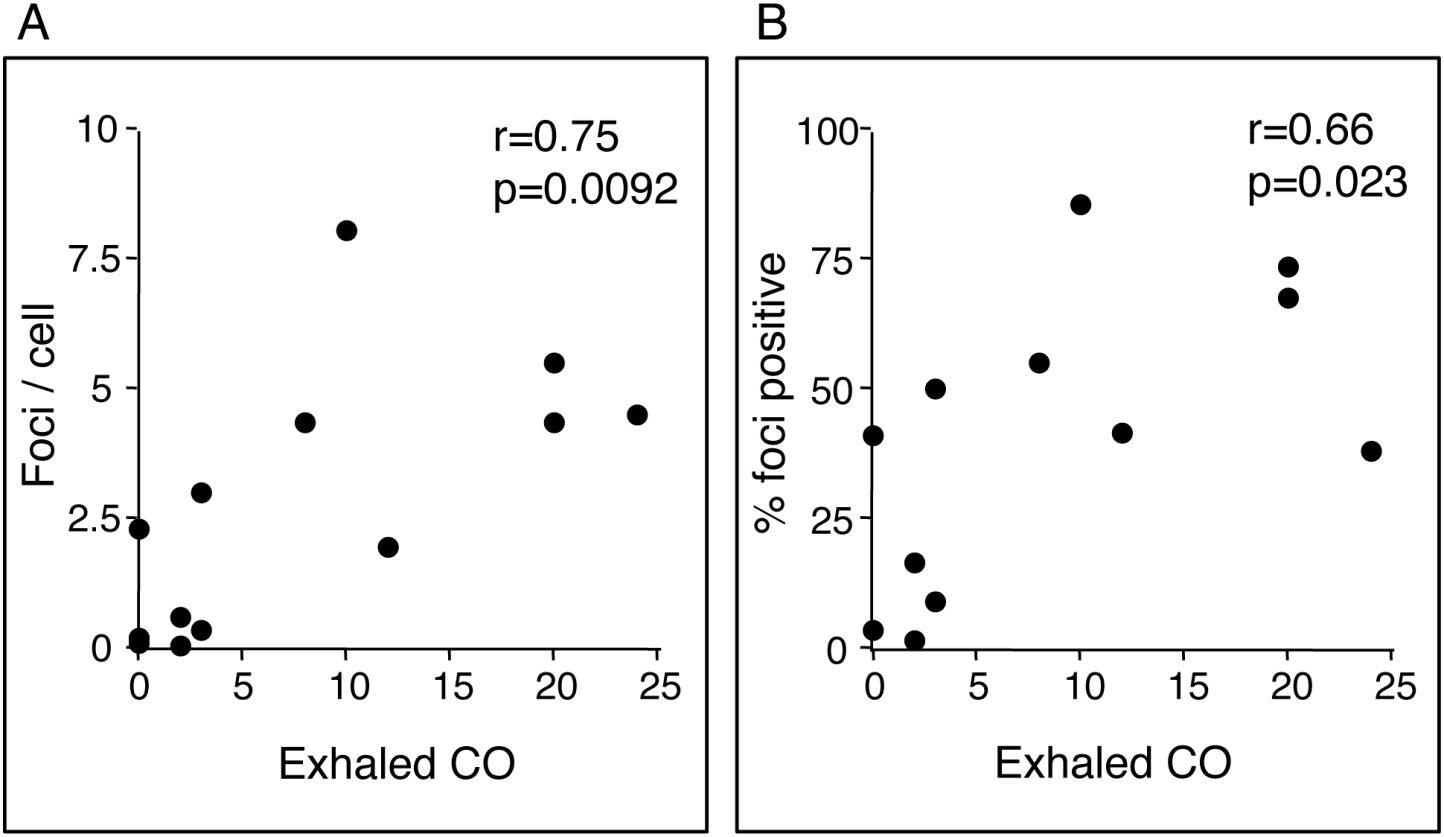
Correlations between foci number per cell (A), percentage of foci positive cells (B), and exhaled carbon monoxide (CO). The Spearman's rank-correlation test was used to assess the relationship between breath CO and γ-H2AX foci number.

The γ-H2AX foci number/cell was significantly lower after smoking cessation when compared with that before smoking cessation (median, 0.28/cell; IQR, 0.12–0.53 vs. 4.43/cell; IQR, 4.36–5.24, p = 0.0277; Fig. 4A). Similarly, the percentage of γ-H2AX foci-positive cells was lower after smoking cessation when compared with that before smoking cessation (median, 6.2%; IQR, 3.5–14.7 vs. 61.2%; IQR, 44.9–72.1, p = 0.0277; Fig. 4B). A strong correlation was noted between the γ-H2AX foci number/cell and exhaled CO (Fig. 5A) and between the percentage of γ-H2AX foci-positive cells and exhaled CO (Fig. 5B) (*r = *0.75, p<0.01 and *r = *0.66, p<0.05, respectively). These results indicate that smoking itself, rather than other factors, induced DSBs and that smoking cessation for 1 month resulted in a decrease in DSBs to a level comparable to that seen in non-smokers.

## Discussion

DSBs are one of the most severe forms of DNA damage and are biologically important. Upon induction of DSBs, a series of mechanisms, termed the DDR, are activated. DDR detects DNA damage via damage sensors, signals their presence by transducers and mediators, and then promotes their repair [15]. Ataxia telangiectasia mutated (ATM), the master regulator of the DDR, senses the chromatin changes elicited by DNA damage, which in turn causes ATM activation. The ATM and other kinases phosphorylate H2AX on the Ser139 residue to form γ-H2AX [16]. γ-H2AX functions as a coordinator of DDR by recruiting specific proteins and providing a binding site for the downstream signaling molecules. Thus, γ-H2AX is an efficient coordinator of DDR [17].

This study focused on DSBs among several types of DNA damage, because DSBs are intrinsically more difficult to repair accurately when compared with other types of DNA damage. In adults, most cells are terminally differentiated and are in the non-dividing state. For these cells, nonhomologous end-joining (NHEJ) is the mechanism whereby DSBs are repaired, because the other repair system (homologous recombination) is generally restricted to the S and G2 phases. NHEJ does not rely on homologies between the two recombining ends; rather, the two ends are simply ligated together. Thus, small sequence deletions are introduced by NHEJ, and DSBs are the hardest type of damage to repair accurately [4]. If DNA damage is not accurately repaired, a sustained DDR results in the inhibition of transcription, replication or chromosome segregation, and leads to cell apoptosis or cell senescence. Mutations and chromosome aberration result in cancer formation. In addition, there is experimental evidence supporting the causal relationship between DSBs and chromosomal aberrations and gene mutation [18].

In the present study, we demonstrated that γ-H2AX and 8-oxo-dG staining is increased in human atherosclerotic plaques, suggesting that DSBs are present in human atherosclerotic plaques and that they co-localize with oxidative DNA damage. Mahmoudi et al. also detected DSBs in human atherosclerotic plaques [19]. In addition, we confirmed that oxidative stress causes DSBs in cultured vascular cells. These data suggest that DSBs are involved in the pathogenesis of atherosclerosis, especially when induced by oxidative stress. Indeed, Werner syndrome arises from abnormalities in the Werner syndrome protein, which is involved in the repair of DSBs, and patients with this syndrome develop atherosclerotic disease at a young age [5]. The molecular mechanisms of the progeroid syndromes support the hypothesis that increased DNA damage, especially DSBs, play a pivotal role in the pathogenesis of atherosclerosis.

Based on the findings showing DSBs presence in human atherosclerotic plaque, we hypothesized that cigarette smoking, one of the major cardiovascular risk factors, causes DSBs. There are several studies on the link between smoking and γ-H2AX formation [7]–[10]. Most of those studies are *in* *vitro* experiments showing that cigarette smoke induces γ-H2AX formation in cultured cells. There is one human study that observed a marked increase in γ-H2AX-positive cells in the placentae from smokers [10]. However, none of these studies showed reversibility of the DSBs in response to smoking cessation within individual subjects. In addition, a strong correlation was noted between the amount of γ-H2AX foci and exhaled CO levels. Thus, this is the first study to demonstrate that smoking increases DSBs in circulating mononuclear cells, that the extent of DSBs depends on the amount of smoking, and that smoking cessation results in reversal of formed DSBs to levels comparable to those seen in non-smokers. Most importantly, the results of the present study underscore the importance of encouraging patients in their efforts to stop smoking.

Mortality in patients with coronary artery disease decreases as early as 2 years after cessation of smoking [20]. Further, endothelial function is disturbed in smokers, and cessation of cigarette smoking rapidly restores endothelium-dependent vascular dilation [21]. Resolution of DNA damage in vascular cells may be one explanation for the beneficial effects of smoking cessation.

Initially produced DNA lesions are processed into DSBs under certain condition. A variety of types of DNA damage, such as oxidation of bases, alkylation of bases, abasic sites, bulky adducts, mismatched base pairs and single strand breaks, are generated during normal cell metabolism and in response to exogenous chemical and biological agents. The oxidized or alkylated bases and abasic sites are subject to base-excision repair. During the repair process, damaged bases are removed, and an apurinic/apyrimidic (AP) site is formed. Removal of AP sites by AP endonucleases or AP lyases, in turn, involves the generation of single strand breaks. Furthermore, persistent single strand breaks can be a source of DSBs via replication fork collapse. Bulky adducts and mismatched base pairs might be converted to DSBs during the repair process by nucleotide-excision repair and mismatch repair, respectively [22], [23]. Thus, the DSBs observed in smokers’ MNCs may arise from other DNA damage, at least in part, while cigarette smoking can directly produce DSBs.

In studies analyzing other types of DNA damage, some studies have shown higher DNA adduct levels in target tissues or leukocytes in smokers relative to those in non-smokers [24]–[26], while others reported no difference in DNA damage between smokers and non-smokers or even lower levels of DNA damage in smokers than in non-smokers [27], [28]. These conflicting results may be related to the type of DNA damage and the methodology employed. The technique adopted in the present study was a direct method of assessing in vivo DSBs and was simple to perform. Further, this methodology demonstrated a marked difference in the degree of DSBs between smokers and non-smokers, a strong correlation between the degree of DSBs and tobacco exposure, and a sharp decrease in DSBs in response to smoking cessation, all of which suggest that this technique is reasonably sensitive and quantitative. In fact, this technique may be a useful and minimally invasive method to estimate DNA damage in response to other noxious stimuli, such as medical or accidental irradiation or mutagenic drugs.

With regard to the limitations of this study, the amount of DSBs in circulating MNCs was not compared with that in relevant target tissues. Therefore, it is not clear whether smoking increases DSBs in the cells of arterial wall from this study. However, some studies have described a correlation between the numbers of DNA adduct in leukocytes and lungs in smokers [29]. We used DNA damage in MNCs as a surrogate marker for that in vascular cells, because MNCs are exposed to the bloodstream in a manner similar to that of vascular cells. In fact, oxidative stress-induced γ-H2AX formation was comparable in MNCs and cultured vascular cells. However, the DNA damage in MNCs may also influence the pathogenesis of atherosclerosis. Indeed, accumulation of DNA damage is generally accepted to be a cause of aging, and gene expression changes and immune response is increased in aged MNCs [30]. Thus, MNCs that have DNA damage may contribute to the acceleration of atherosclerosis.

Accumulation of DNA damage activates DDR systems and forces the cells to undergo apoptosis or senescence, both of which are features seen in atherosclerotic plaques [31]. Apoptosis of macrophages results in formation of a necrotic core, while apoptosis of vascular smooth muscle cells increases the vulnerability of plaques to rupture [32], [33]. Cell senescence fuels inflammation and probably accelerates atherosclerosis [34]–[36]. Further study to determine if there is a causal relationship between DSBs and atherosclerosis formation will contribute to the prevention of atherosclerotic cardiovascular disease.

In conclusion, the present study demonstrated that cigarette smoking increases DSBs in human MNCs. Most importantly, smoking cessation reverses DSBs to levels comparable to those seen in non-smokers. These data reinforce the importance of smoking cessation. Furthermore, as the technique adopted in the present study is simple to perform and reasonably sensitive and quantitative, this technique may be a useful minimally invasive method to estimate DNA damage in response to other noxious stimuli, such as medical or accidental irradiation or mutagenic drugs.
